# Supplementary material for: Epitaxial bulk acoustic wave resonators as highly coherent multi-phonon sources for quantum acoustodynamics
Source: Nat Commun. 2020 May 8;11:2314. doi: 10.1038/s41467-020-15472-w (PMC7210958; doi:10.1038/s41467-020-15472-w)
Supplement: Supplementary file 1 — Supplementary Information [file 41467_2020_15472_MOESM1_ESM.pdf]

Supplementary Information

Gokhale et al.

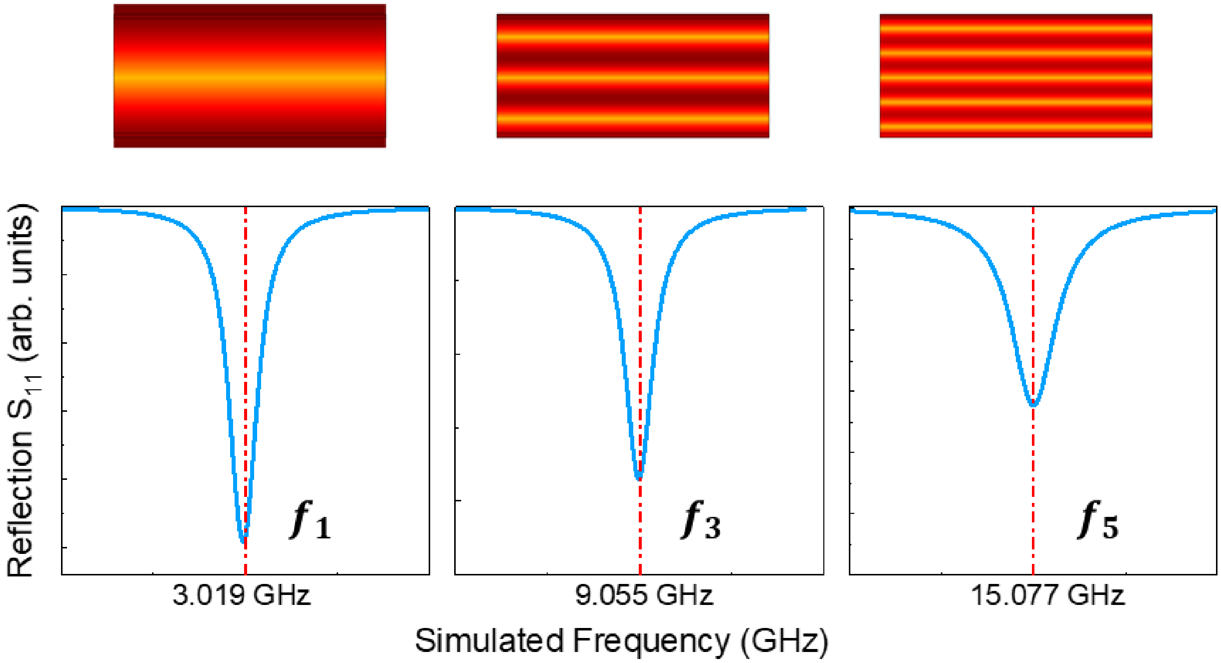

Supplementary Figure 1: Simulated frequencies of the transducer envelope modes. Finite element simulations of a hypothetical semi-infinite FBAR made from the Al/GaN/NbN heterostructure used for the epi-HBAR. Displacement mode shapes and reflection spectra are shown here. Note that the FBAR does not have the SiC substrate. This results in a thickness-mode resonator with free-free mechanical boundary conditions with the first three modes of resonance shown. Only odd multiples of the fundamental thickness mode are allowed by the free-free boundary conditions. In the epi-HBAR, these three transducer modes are loaded by the substrate, broadening them considerably and forming the transducer envelope modes for the epi-HBAR.

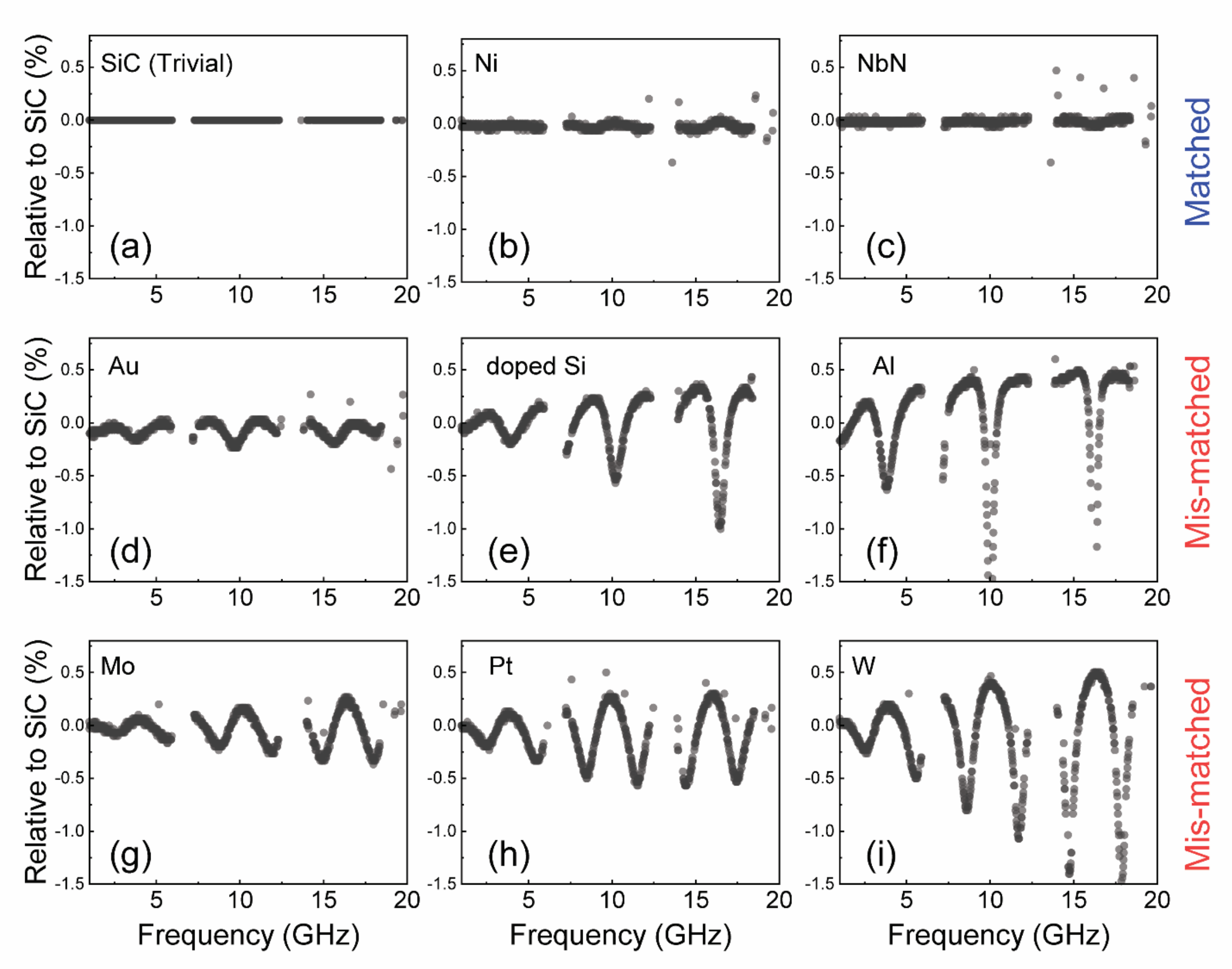

Supplementary Figure 2: Effect of bottom electrode material on acoustic impedance matching. Numerically calculated FSR distributions for epi-HBARs with variable bottom electrode materials, normalized to the FSR distribution of the hypothetical situation with a SiC electrode. All other parameters are kept constant. The bottom electrode materials are indicated in each panel. (a) The hypothetical SiC bottom electrode is perfectly matched to the SiC substrate. (b) Ni, and (c) NbN, are good candidates for the bottom electrode with acoustic impedance matching to SiC across a broad frequency range. In contrast, (d)-(i) are examples of mismatched bottom electrode materials, with low stiffness materials (such as Al) or dense materials (such as W and Pt) presenting a large acoustic mismatch to SiC. The FSR distribution is calculated using the HBAR model described by Zhang, Cheeke, and Hickernell [1-3].

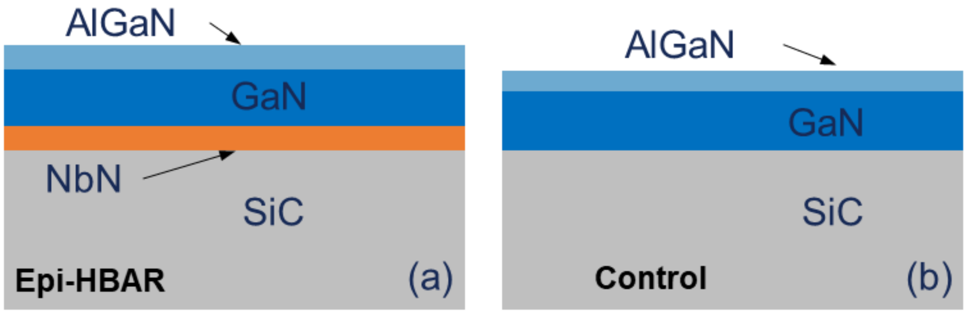

Supplementary Figure 3: Schematic cross-sections of the heterostructures. Schematic cross-sections (not to scale) of (a) the epitaxial heterostructure used for the epi-HBARs, and (b) the epitaxial heterostructure of the control sample without NbN.

Supplementary Table 1: **Epitaxial heterostructure composition and properties.** The alloy compositions of the AlGaN barrier layer, layer thickness, and electronic properties of the heterostructure used for the epi-HBARs, and the heterostructure of the control sample without NbN

Parameter	Symbol	Material	Epi-HBAR	Control
Thickness	*t (nm)*	AlGaN	25	25
Al fraction	Al (%)		29	35
Thickness	*t* (nm)	GaN	1200	1200
Carrier Density	n_{sh} (×10^{12}cm^{-2})		6.9	13.6
Mobility	μ (cm^2/V-s)		1000	900
Sheet Resistance	R_{sh} (Ω/□)		920	500
Thickness	*t* (nm)	NbN_x	50	*N/A*
Sheet Resistance	R_{sh} (Ω/□)		9	*N/A*

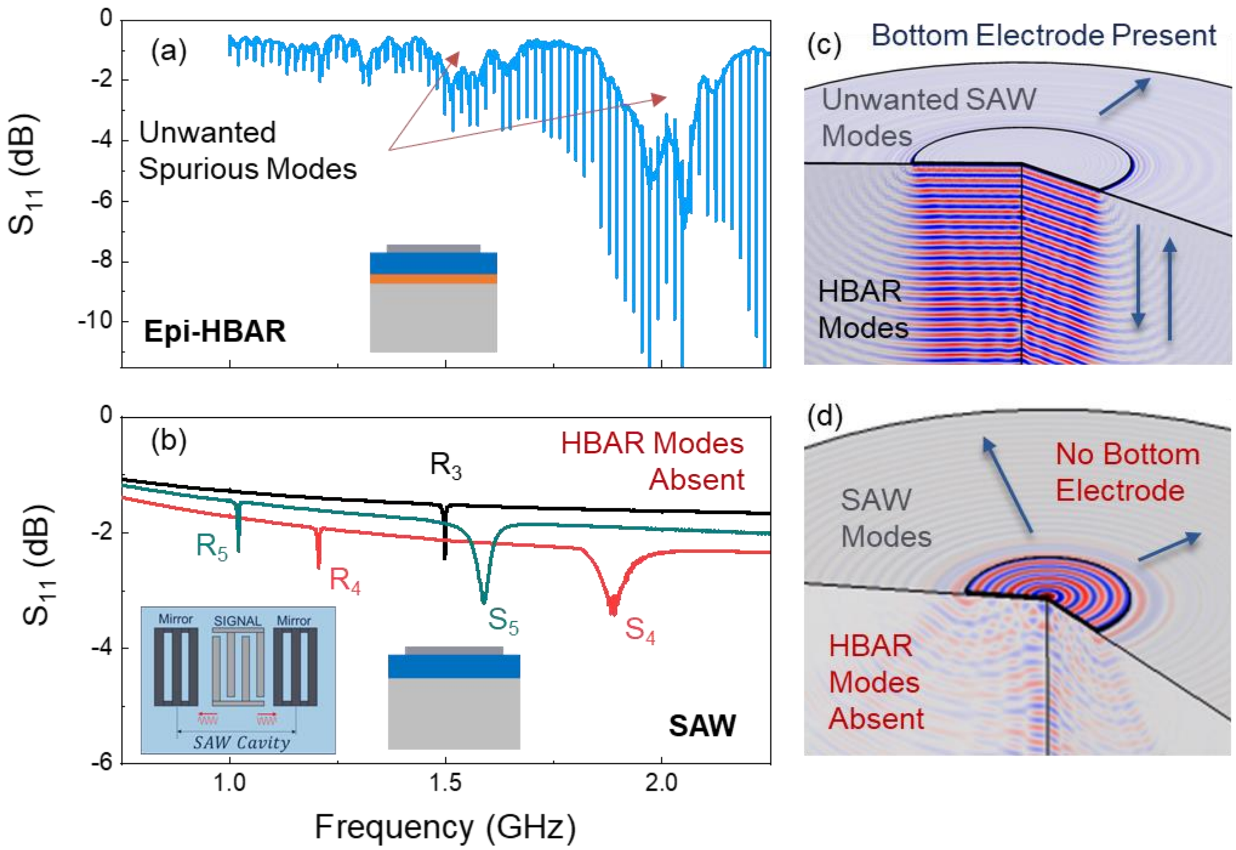

Supplementary Figure 4: The importance of the bottom electrode for transducing longitudinal phonon modes. A low frequency ($f < 2.5$ GHz) comparison between (a) the epi-HBAR (GaN/NbN/SiC heterostructure), and (b) SAW devices made on a control sample (GaN/SiC heterostructure) grown under similar conditions, but without the NbN conducting bottom electrode. Specifics of the control sample are provided in . The key difference between the two heterostructures is that when the bottom electrode is present, the metal-piezoelectric-metal heterostructure acts as a vertical transducer and generates longitudinal modes that can be pumped into the phonon cavity (substrate). Spurious modes in the low frequency ($f < 2.5$ GHz) region can be due to unwanted shear waves or SAW waves in the epi-HBAR. The various SAW modes in (b) correspond to Rayleigh (R_Λ), and Sezawa (S_Λ), where the SAW wavelength is given by $\Lambda \in [3,4,5]$ µm. (c) Axisymmetric finite element analysis for the GaN/NbN/SiC heterostructure qualitatively confirms generation of the HBAR modes in the vertical direction due to the presence of the Al/GaN/NbN piezoelectric transducer (and subsequent trapping in the phonon cavity). (d) In the absence of the bottom electrode (control sample), HBAR modes are not actuated. The circular electrodes are a feature of the axisymmetric simulation, and not intended to model the exact electrode dimensions of fabricated device. A complete analysis of electrode shape and size dependence on HBAR performance is beyond the scope of this work.

Supplementary Table 2: Anharmonic phonon loss regimes and material properties used for calculation. The anharmonic phonon losses pose the ultimate theoretical energy loss limits for BAW devices such as HBARs operating at low frequencies (Akhieser regime) and high frequency (Landau-Rumer regime)

Loss regime	Frequency Range	$f \times Q$ Upper Limit
Akhieser	$\omega\tau_{th} \ll 1$	$f \times Q_{anh} = \left(\frac{\rho v^2}{2\pi C_v \gamma^2 \tau_{th}}\right)\frac{1}{T}$
Landau-Rumer	$\omega\tau_{th} > 1$	$f \times Q_{anh} = \left(\frac{15\rho v^5 h^3}{\pi^5 \gamma^2 k_b^4}\right)\frac{f}{T^4}$
**Material properties of 4H- SiC**		
**Quantity**	**Symbol**	**Value used**
Temperature	T	variable
Mass density	ρ	3210 kg/m^3
Acoustic Velocity	v	13300 m/s
Volumetric heat capacity	C_v	1.92×10^6 J/m^{3}K
Thermal phonon lifetime	τ_{th}	10^{-12} s
Grüneisen parameter	γ	0.8

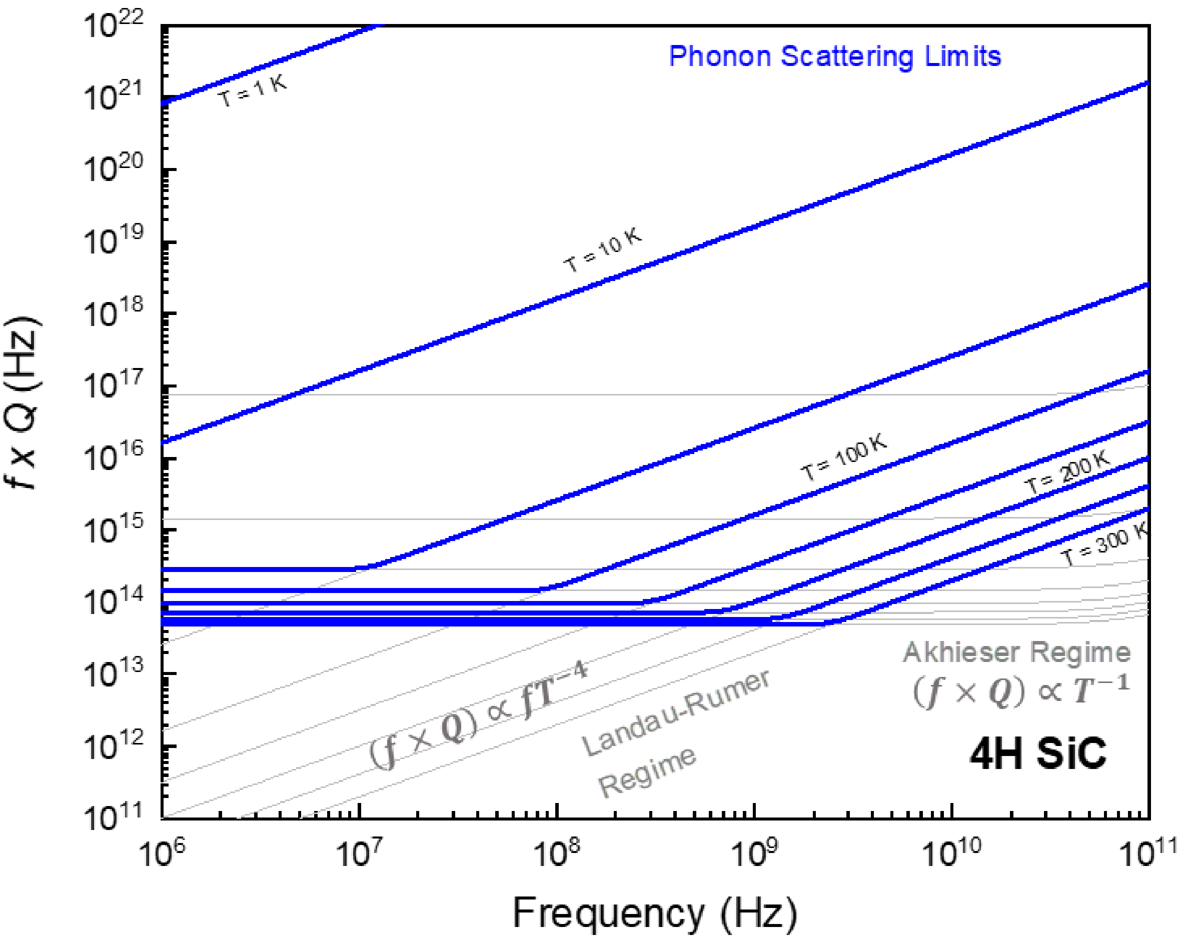

Supplementary Figure 5: Anharmonic phonon scattering for the phonon cavity. Theoretical model for the anharmonic phonon scattering limits (in terms of maximum $f \times Q_{anh}$) for 4H-SiC, calculated from published material properties from Supplementary Table 2, and following the Akhieser and Landau Rumer phonon scattering regimes at various temperatures [4-8]. These models do not take into account the effects of any other loss mechanism.

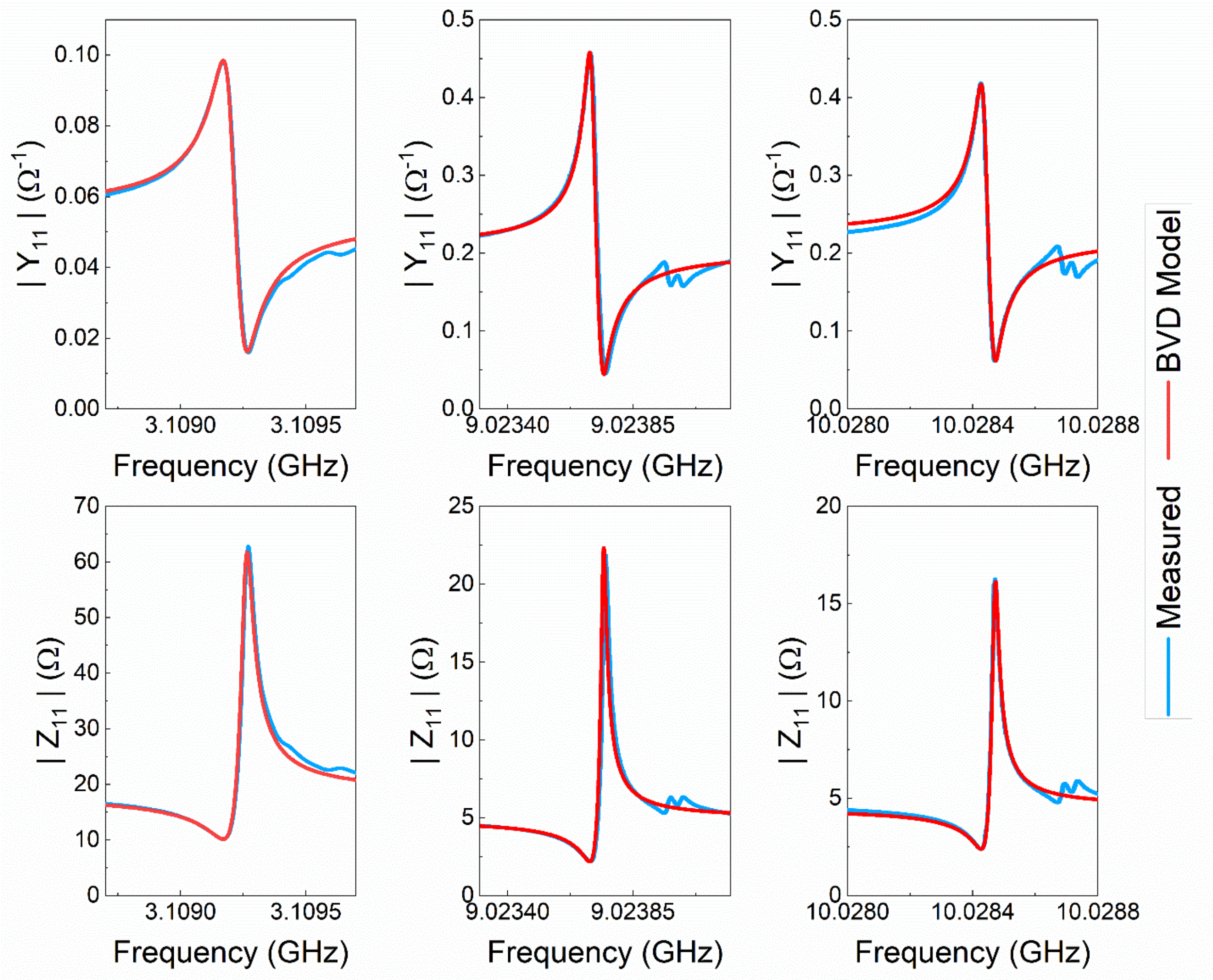

Supplementary Figure 6: Impedance and admittance fits for selected epi-HBAR modes. Measured (blue) and modeled (red) admittance ($|Y_{11}|(\Omega^{-1})$) and impedance $|Z_{11}|(\Omega)$) plots for the three representative epi-HBAR modes (m = (164, 476, and 529)) shown in Fig. 3(e)-(g).

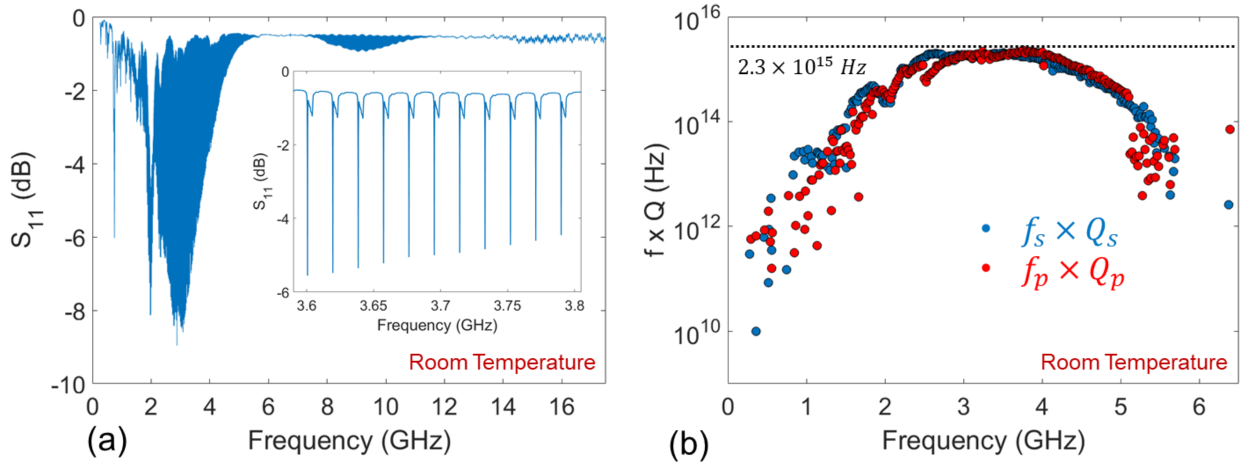

Supplementary Figure 7: Room temperature performance of the epi-HBAR. (a) Room temperature measurements showing the microwave reflection spectrum ($S_{11}(\omega)$) for the epi-HBAR. Inset shows a magnified section of the spectrum with individual epi-HBAR phonon modes clearly visible. (b) Measured room temperature $f \times Q$ products for the epi-HBAR. While the performance at room temperature is worse than the 7.2 K measurements (as expected), the maximum value of 2.3×10^{15} Hz at 3.75 GHz is significantly higher than sputter-deposited HBARs operated at room temperature with reported $f \times Q$ products of $\approx 2\times10^{14}$ Hz [9-11].

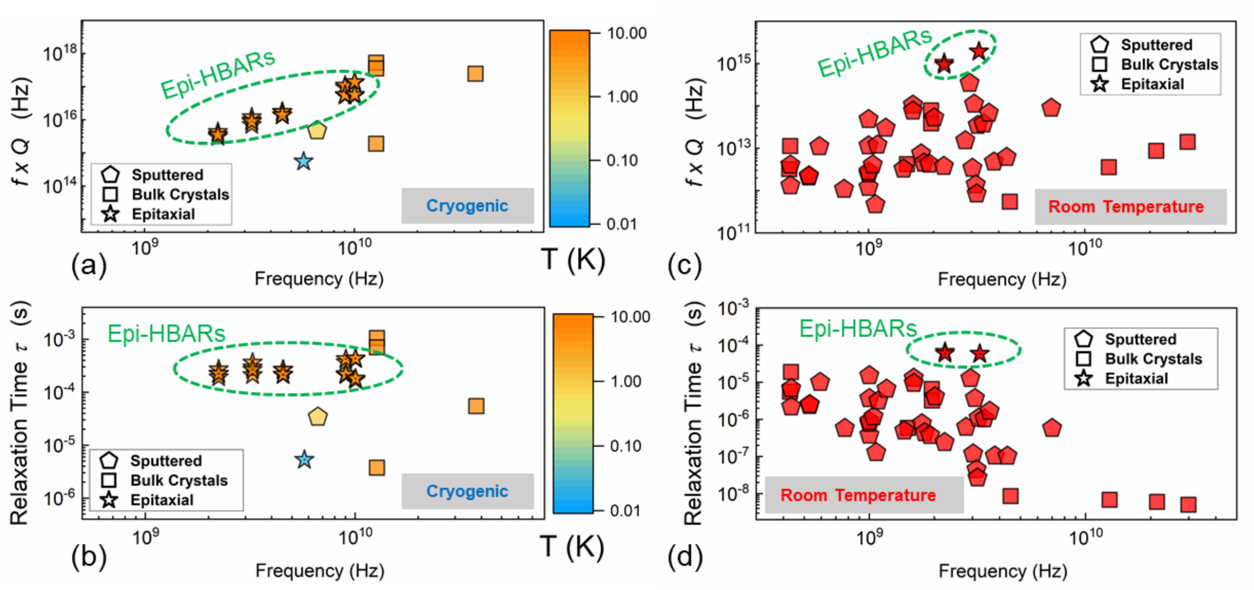

Supplementary Figure 8: Superior performance of epi-HBARs at both cryogenic and room temperature. A comparison between measured $f \times Q$ product and τ for epi-HBARs from this work and other reported values in literature, for specific temperatures or temperature ranges of interest. The data are compared at (a)-(b) cryogenic temperature ($T \leq 10\ K$), and (c)-(d) at room temperature. Note that the color fill for data in (a) and (b) corresponds to measurement temperature on a logarithmic scale from 0.1 (blue) to 10 (orange).

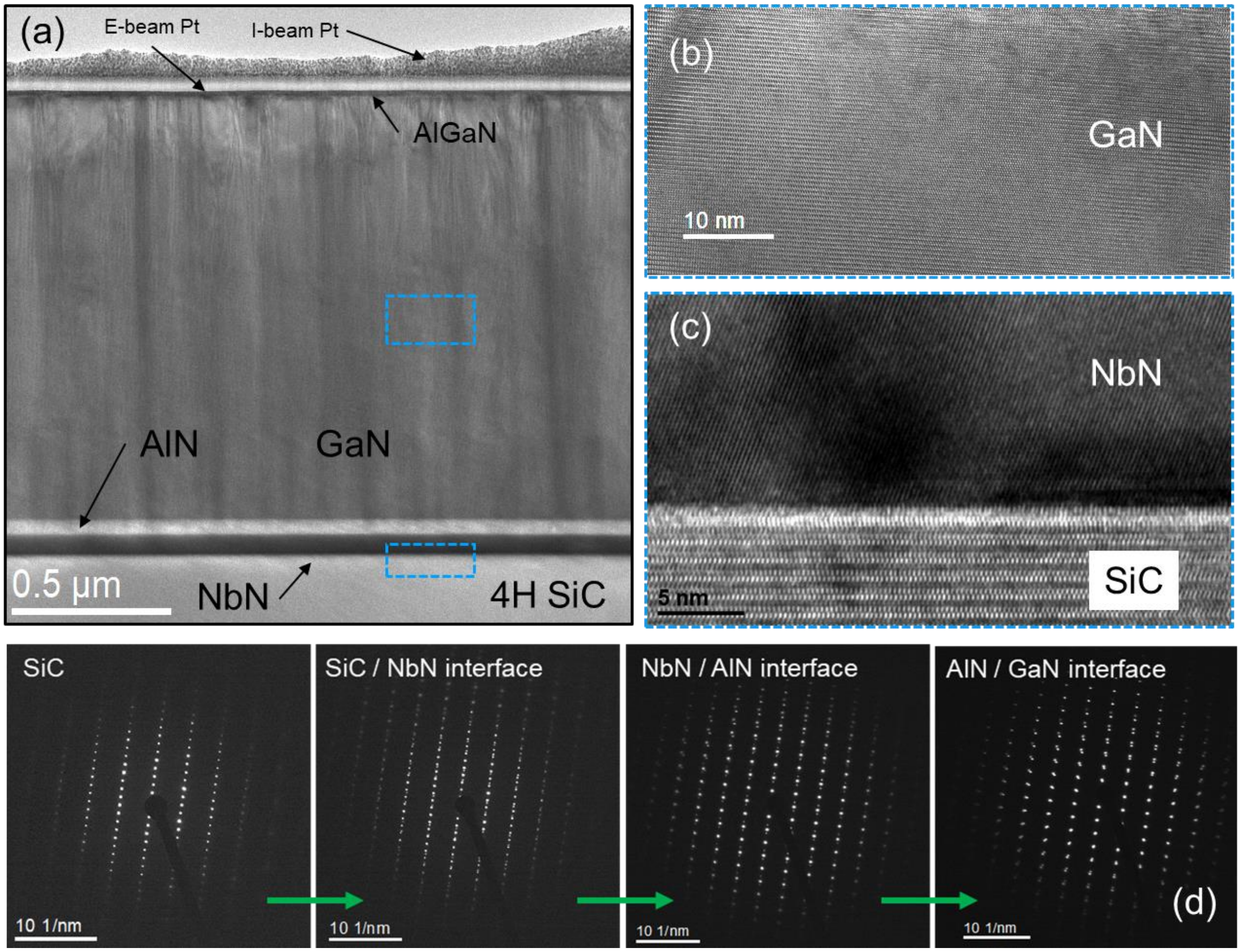

Supplementary Figure 9: Electron microscopy analysis of the epi-HBAR heterostructure. (a) TEM image of the AlGaN/GaN/AlN/NbN/SiC heterostructure used for making the epi-HBAR. Note that the AlGaN barrier is etched down prior to fabricating the HBAR, but can be used elsewhere on the wafer for co-integrating AlGaN/GaN HEMT based electronics. Pt layers on the top of the film are a feature of the TEM sample preparation process, and are not part of the epi-HBAR. (b) HRTEM image of the thick GaN layer shows a clean regular lattice with no visible defects. The image in (b) is brightness/contrast adjusted for visual clarity. (c) HRTEM image of the smooth and regular interface between the SiC substrate and the NbN bottom electrode. The NbN/SiC interface is crucial to the operation of the epi-HBAR since it defines the boundary between the active piezoelectric transducer and the passive substrate. (d) SAED images of the various interfaces in the heterostructure indicate a highly oriented epitaxial heterostructure, with low lattice mismatch across successive layers and with a well-aligned piezoelectric axis. SAED imaging was performed with a 100 nm aperture.

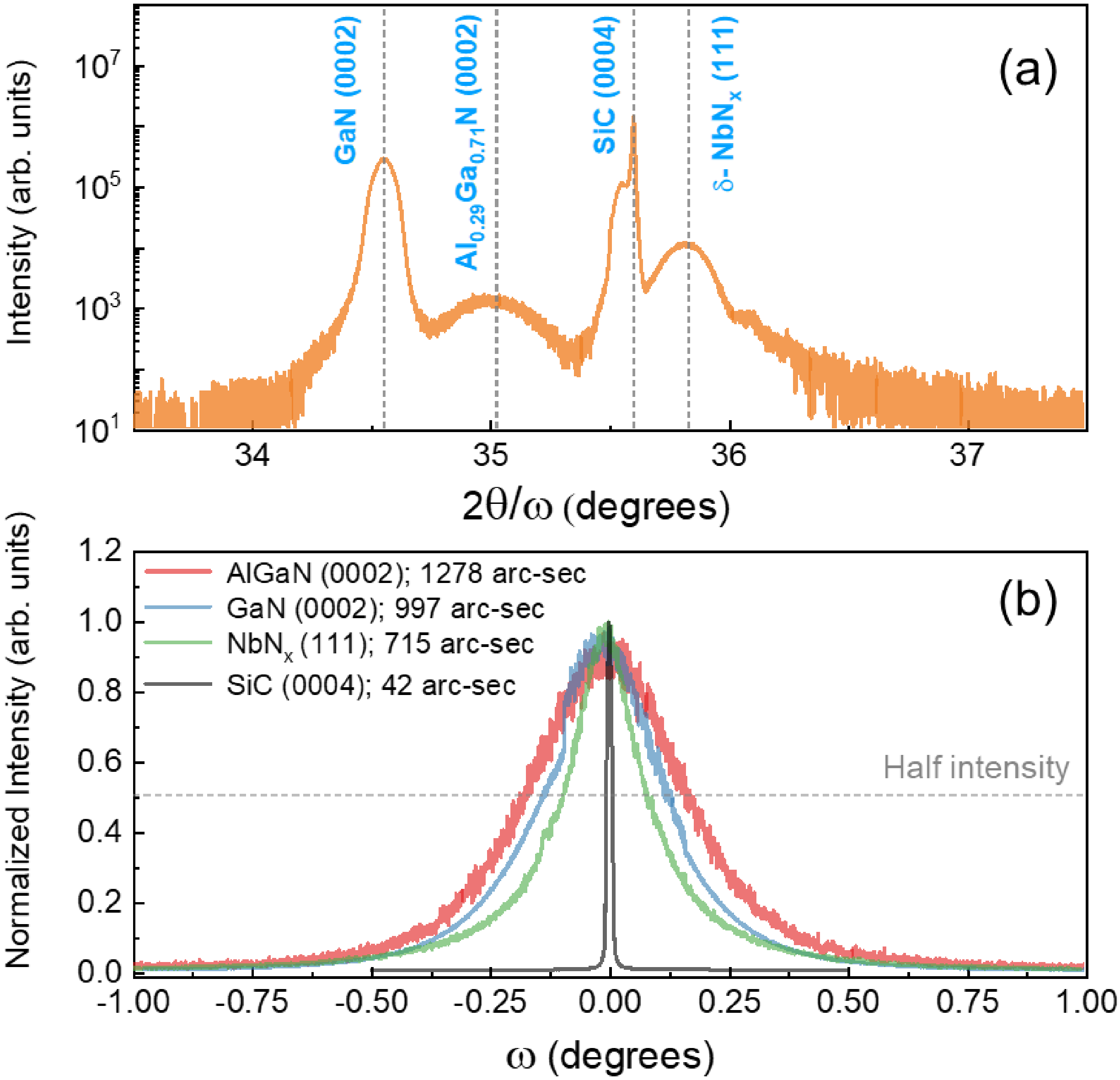

Supplementary Figure 10: X-ray diffraction (XRD) analysis of the epi-HBAR heterostructure. (a) XRD analysis of the AlGaN/GaN/AlN/NbN/SiC heterostructure confirms the material peaks for GaN (0002), $Al_{0.29}Ga_{0.71}N$ (0002), δ −NbN (111), and SiC (0004). The cubic phase δ −NbN has a lattice constant of 0.31 nm, which is between the lattice constant values for 4H-SiC and AlN or GaN [12]. (b) Rocking curves of the constituent material layers show FWHM values ranging 42 arc-sec for the bulk SiC substrate, to 715 arc-sec and 997 arcsec for the NbN_x and GaN films, respectively.

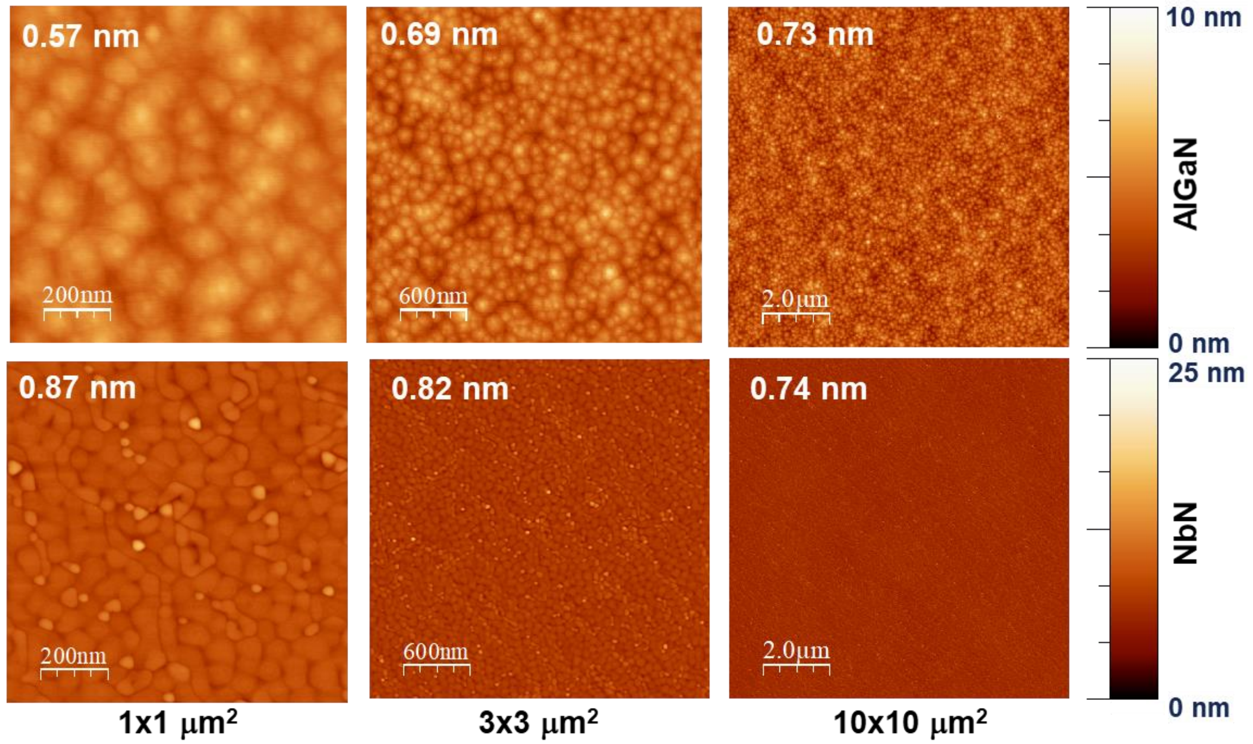

Supplementary Figure 11: Atomic force microscopy (AFM) analysis of the epi-HBAR heterostructure. AFM scans of the surfaces of the NbN and AlGaN layers (respectively, the first and last layers to be grown epitaxially), indicating smooth surfaces with sub-nanometer RMS surface roughness across the entire epitaxial process. AFM images are taken at three scan sizes for each film. Note that the AlGaN layer itself is not part of the epi-HBAR, and is etched away before the top electrode of the epi-HBAR is deposited and patterned. The data on NbN are acquired on a representative NbN/SiC sample with the same NbN thickness (50 nm).

SUPPLEMENTARY REFERENCES

1 Zuoging, W., Yuxing, Z. & Cheeke, J. D. N., Characterization of electromechanical coupling coefficients of piezoelectric films using composite resonators, *IEEE Transactions on Ultrasonics, Ferroelectrics, and Frequency Control* **46**, 1327-1330 (1999).

2 Zhang, Y., Wang, Z., Cheeke, J. D. N. & Hickernell, F. S., Direct characterization of ZnO films in composite resonators by the resonance spectrum method, in *IEEE Ultrasonics Symposium*, 991-994 (1999).

3 Yuxing, Z., Zuoqing, W. & Cheeke, J. D. N., Resonant spectrum method to characterize piezoelectric films in composite resonators, *IEEE Transactions on Ultrasonics, Ferroelectrics, and Frequency Control* **50**, 321-333 (2003).

4 Akhieser, A., On the absorption of sound in solids, *Journal of Physics* **1**, 277-287 (1939).

5 Braginsky, V. B. & Mitrofanov, V. P. in *Systems with Small Dissipation,* (ed Kip. S. Thorne) Ch. 1, 1-42, The University of Chicago Press (1986).

6 Rais-Zadeh, M. *et al.*, Gallium nitride as an electromechanical material, *J Microelectromech S* **23**, 1252-1271 (2014).

7 Gokhale, V. J. & Gorman, J. J., Approaching the intrinsic quality factor limit for micromechanical bulk acoustic resonators using phononic crystal tethers, *Appl Phys Lett* **111**, 013501 (2017).

8 Ghaffari, S. *et al.*, Quantum Limit of Quality Factor in Silicon Micro and Nano Mechanical Resonators, *Scientific Reports* **3**, 3244 (2013).

9 Lakin, K. M., Kline, G. R. & McCarron, K. T., High Q microwave acoustic resonators and filters, in *1993 IEEE MTT-S International Microwave Symposium (IMS),* 1517-1520 (1993).

10 Liu, M., Li, J., Wang, C., Li, J. & Ma, J., Influence of electrodes on the effective electromechanical coupling coefficient distributions of high-overtone bulk acoustic resonator, *Ultrasonics* **56**, 566-574 (2015).

11 Ziaei-Moayyed, M., Habermehl, S. D., Branch, D. W., Clews, P. J. & Olsson, R. H., Silicon carbide lateral overtone bulk acoustic resonator with ultrahigh quality factor, in *IEEE International Conference on Micro Electro Mechanical Systems (MEMS)*, 788-792 (2011).

12 Downey, B. P., Meyer, D. J., Hardy, M. T., Gokhale, V. J. & Jin, E. N., Epitaxial Transition Metal Nitride/III N Alloys for RF Devices, in *GOMACTech*, 592-595 (2019).
